# Description of the Current Da Vinci^®^ Training Pathway for Robotic Abdominal Wall Surgery by the European Hernia Society

**DOI:** 10.3389/jaws.2022.10914

**Published:** 2022-11-30

**Authors:** Maaike Vierstraete, Maarten Simons, Knut Borch, Andrew de Beaux, Barbora East, Wolfgang Reinpold, Cesare Stabilini, Filip Muysoms

**Affiliations:** ^1^ Department of General Surgery, AZ Maria Middelares, Ghent, Belgium; ^2^ Department of Surgery, OLVG Hospital, Amsterdam, Netherlands; ^3^ General Surgical Department, Hernia Center, University Hospital of North Norway, Tromsø, Norway; ^4^ Spire Murrayfield Hospital, Edinburgh, United Kingdom; ^5^ 3rd Department of Surgery, 1st Medical Faculty at Charles University, Prague, Czechia; ^6^ Motol University Hospital, Prague, Czechia; ^7^ Department of Hernia and Abdominal Wall Surgery, Helios Mariahilf Hospital ATOS Klinik Fleetinsel, Hamburg, Germany; ^8^ Dipartimento di Scienze Chirurgiche (DISC), Università Degli Studi di Genova, ITA Policlinico San Martino IRCCS, Genoa, Italy

**Keywords:** hernia, robotic surgery, education, robotic training, inguinal hernia, ventral hernia, robotic abdominal wall surgery

## Abstract

**Background:** Robot assisted laparoscopic abdominal wall surgery (RAWS) has seen a rapid adoption in recent years. The safe introduction of the robot platform in the treatment of abdominal wall hernias is important to safeguard the patient from harm during the learning curve. The scope of this paper is to describe the current European training curriculum in RAWS.

**Methods and Analysis:** The pathway to competence in RAWS will depend on the robot platform, experience in other abdominal procedures (novice to expert) and experience in the abdominal wall repair techniques. An overview of the learning curve effect in the initial case series of several early adopters in RAWS was reviewed. In European centres, current training for surgeons wanting to adopt RAWS is managed by the specific technology-based training organized by the company providing the robot. It consists of four phases where phases I and II are preclinical, while phases III and IV focus on the introduction of the robotic platform into surgical practice.

**Conclusion:** On behalf of the Robotic Surgery Task Force of the European Hernia Society (EHS) we believe that the EHS should play an important role in the clinical phases III and IV training. Courses organized in collaboration with the robot provider on relevant surgical anatomy of the abdominal wall and procedural steps in complex abdominal wall reconstruction like transversus abdominis release are essential. Whereas the robot provider should be responsible for the preclinical phases I and II to gain familiarity in the specific robot platform.

## Introduction

Robot assisted laparoscopic abdominal wall surgery (RAWS) has seen a rapid adoption particularly in the United States. In Europe, the application rate has been slower, hampered by the restrictions and limitations in surgical procedures during the COVID pandemic. The safe introduction of RAWS is important to avoid any negative connotations of robot assisted laparoscopic surgery and to safeguard the patient from harm. It is important that surgeons are properly trained in the use of robotic technology and guided in a safe progressive introduction of the acquired skills into their clinical practice.

The aim of this study was to describe the current training curriculum in RAWS by the Robotic Surgery Task Force of the European Hernia Society (EHS).

## The Trainee

The surgeon starting a pathway in RAWS in their clinical practice will come from different backgrounds in exposure to both abdominal wall surgery and robotic expertise. This baseline experience of the surgeon will likely influence the learning trajectory they would need to follow for a safe and efficient training pathway (Table 1).

**TABLE 1:** Baseline experience of the trainee surgeon for robot assisted abdominal wall surgery.

	Surgical trainee	Open surgeon ^a^	MIS surgeon ^b^	Robotic surgeon ^c^
Hernia specialist ^d^	X	A	B	C
Non-hernia specialist ^e^		D	E	F

^a^ Surgeon performing mainly open hernia surgery with limited practice in laparoscopy.

^b^ Surgeon performing mainly laparoscopic hernia repair.

^c^ Surgeon performing having robotic training and clinical experience.

^d^ Surgeon with a specific hernia interest and substantial case load in hernia repair.

^e^ Surgeon without previous specific hernia interest or substantial case load in hernia repair.

Surgeons in terms of their experience at the time of RAWS training can be divided into “hernia specialist” or “non-hernia specialist.” This is of course a difficult discriminator. Most general surgeons perform hernia repairs, but we know from the Danish nation-wide data that many surgeons only perform a low number of hernia operations (1). It is difficult to put an exact number to the volume of procedures per year a surgeon must perform to qualify as a hernia specialist. The ACCESS project tried to identify criteria for hernia centres and surgeons: “*A general surgeon can be trained to become a specialist hernia surgeon by mastering the learning curve of all open and laparo-endoscopic hernia procedures that are recommended in the guidelines and should additionally implement and fulfil the other requirements for a hernia centre and perform a minimal yearly caseload*.” (2).

The caseload number required to qualify as a hernia specialist was not specified by the ACCESS group. It was suggested that: “*... to perform a significantly higher case volume in all types of hernia surgery compared to an average general surgery department in their country.*” The volume required in the German program for accreditation/certification of a high-level hernia centre is at least 250 hernia operations per year (3). In the Italian Society of Hernia and Abdominal Wall Surgery for the third level classification “High Specialization for Abdominal Wall Surgery,” the volume requirements for inguinal hernia repair are 150 procedures with 20 recurrent or scrotal hernias and 50 abdominal wall repairs with 20 complex cases (4). Little evidence is put forward to justify these numbers.

Recently the European Board of Specialists (EBS) and Union of European Medical Specialists (UEMS) have created the subspeciality of Abdominal Wall Surgery (AWS) in collaboration with the EHS. Surgeons can apply for the title of Fellow of the EBS/AWS by passing an exam organised in proximity of the annual congress of the EHS. The EBS has defined eligibility requirements for UEMS AWS qualification which can be found on the UEMS website.^1^ A total combined number of 800 credits points have to be acquired which correlates with about 200 inguinal hernia repairs, 50 primary ventral hernia repairs and 50 incisional and complex hernia repairs, in addition to 200 CME points from training courses.

Firstly, the abdominal wall surgery experience of the RAWS “trainee” surgeon. This will be partly numbers of procedures (groin/ventral) although competency-based training is increasingly being utilized in surgical training assessment. Secondly, the prior experience of the RAWS “trainee” surgeon with the robotic platform. Learning a new operation whilst using new technology will overwhelm the mental and dexterous skills of most surgeons. The potential categories of a RAWS “trainee” surgeon are described in Table 1.

A key element to training in RAWS is regular and frequent access to the robotic platform in the start-up phase, preferably on a weekly basis during the initial case series.

## The Learning Curve for Robot Assisted Abdominal Wall Surgery: Literature Review

Several early adopters of RAWS have evaluated the learning curve effect in their initial case series. Most researchers have used the operative time (OT), measured as the time from first skin incision to the last skin suture, as the primary outcome to study the learning curve. It is postulated that the learning curve has been achieved when the OT has reached a plateau. Although it is clear that there is more to a learning curve than simply the OT evolution, other metrics to evaluate the learning curve are more difficult to measure and assess within clinical practice.

### Groin Hernias

Robot assisted laparoscopic groin hernia repair is often considered the ideal starting operation in a robotic hernia surgery program (5–7). The repair of simple groin hernias is commonplace in a general surgery practice. And laparoscopic transabdominal preperitoneal prosthetic repair (TAPP) or totally extraperitoneal prosthetic repair (TEP) is performed by many surgeons with an interest in abdominal wall surgery. Therefore, we consider the robot assisted laparoscopic repair of simple groin hernias (rTAPP) as the ideal initial case choice. It allows for achieving proficiency with the use of the robotic platform in a standardized well mastered procedure requiring docking, dissection, mesh handling and suturing skills. In addition, the relative short length of the procedure, allows the team to plan several cases in one operating day, facilitating repetitive training in the preparation of the robotic platform, draping, docking and safe instrument introduction.

In a series of their initial RAWS cases (8) acknowledge a significant decrease of OT in the second half of the first 25 bilateral rTAPP operations performed by an experienced senior surgeon (*trainee type B*). (9) found that for a senior surgeon experienced in both laparoscopic hernia repair and robotic gastrointestinal surgery (*trainee type C*) 35 cases seemed to be needed to achieve optimum OT for rTAPP. Ebeling et al (10) described a dedicated training program for residents (*trainee type X*) for rTAPP of groin hernias. They propose to discriminate an rTAPP operation in 4 segments: flap creation, hernia reduction, mesh placement and flap closure. Trainees were evaluated with grading according to GEARS (Global Evaluative Assessment of Robotic Skills) and the Zwisch scale. To receive a “Certificate of Equivalency” from Intuitive Surgical Inc the trainee must complete 10 bedside assisted cases and 20 console cases (with at least 50% of the surgical time performed by the trainee). This can be used by credentialing entities in hospitals. Ebeling et al. (10) however state that these cut-off values are rather arbitrarily chosen, and their data indicate that residents demonstrate significantly higher competency scores after 30 robotic cases as a primary console surgeon. Muysoms et al. (7) analysed the evolution of OT for their first 50 cases of rTAPP for bilateral or unilateral groin hernias by a robotic naïve surgeon with extensive experience in MIS hernia repair (*trainee type B*). They observed a rapid reduction in operative time during the learning curve and after 20 to 25 cases the operative time to perform a rTAPP equalled the operative time to perform a conventional laparoscopic TAPP, both for unilateral and for bilateral groin hernia repairs. No complications related to the introduction of robotic-assisted laparoscopic groin hernia repair were observed. Kudsi et al. (5) have used the methodology of cumulative sum analysis (CUSUM) to evaluate the OT of rTAPP and the learning curve. They reported that regardless of bilateral or complex patient indications for rTAPP, the OT and surgical site events (SSE) rates gradually decreased after completing 138 procedures. Proietti et al. (11) showed that rTAPP performed by experienced laparoscopic surgeons (*trainee type B*) had a learning curve which required 43 inguinal hernia repairs to achieve 90% proficiency and to significantly reduce the OT. When looking at the unilateral rTAPP alone, 25 cases were necessary to complete the learning curve. Prabhu et al. (12) published a randomized controlled trial on rTAPP in 102 patients with inguinal hernia, with no significant differences in operative outcomes at 30 days found between patients who received robotic inguinal hernia repair and those who received laparoscopic inguinal hernia repair in terms of postoperative pain, health-related quality of life, mobility, wound morbidity, or cosmesis. The robotic approach resulted in increased operative time, cost, and surgeon frustration, without discernible ergonomic benefit for surgeons. We think there are some surprising results and limitations to this study. The technique was significantly different between conventional laparoscopic cases (TAPP) and robotic assisted laparoscopic cases (rTAPP). In the TAPP cases a tacker fixation device was used for mesh fixation and closure of the peritoneum, while for rTAPP sutures were used, which likely accounts for the longer OT for rTAPP. We also think that the assumption of the authors is incorrect, as they guarantee that no learning curve effect was present in surgeons who performed at least 25 surgeries with the robotic platform and 25 with conventional laparoscopy. Since the required 25 robotic cases were not necessarily groin hernia repairs and the large gap between the experience of conventional laparoscopic cases versus robotic cases, we do think that several of the outcomes are probably related to a learning curve effect and familiarity with the specifics of using the robotic platform for rTAPP of groin hernias. An overview of the studies is listed in Table 2.

**TABLE 2:** Overview of studies reporting on the learning curve for RAWS.

		Study	Outcome	Trainee type	Learning curve
Inguinal hernia					
	Ephraim (8)	Retrospective study	Operative time (OT)	B	Significant decrease in OT in the second half of the first 25 bilateral rTAPP surgeries
	Aghayeva (9)	Retrospective CUSUM analysis	OT	C	35 Cases to achieve optimum OT for rTAPP
	Ebeling (10)	Prospective study	Competency score	X	Significantly higher competency scores after 30 robotic cases as a primary console surgeon
		GEARS and Zwisch scoring			
	Muysoms (11)	Prospective study	OT	B	OT for rTAPP equals the OT for laparoscopic TAPP after 20 to 25 cases
	Kudsi (6)	Retrospective CUSUM analysis	OT	B	OT and SSE rates decreased after 138 procedures, regardless of bilateral or complex status
			Surgical site events (SSE)		
	Proietti (13)	Retrospective CUSUM analysis	OT	B	Significantly reduced OT and 90% proficiency after 43 rTAPPs
					For unilateral rTAPP, 25cases were necessary to complete the learning curve
Ventral hernia					
rIPOM	Kudsi (14)	Retrospective CUSUM analysis	OT	C	Consistent and gradual decrease in OT after 36 cases
			Improving outcomes		Improving outcomes after 55 cases
rTAPP	Kudsi (15)	Retrospective CUSUM analysis	OT	C	Steadily decrease in OT after 46 cases
			Good quality repair (i.e. maintenance of the integrity of the peritoneal flap)		Good quality repair, gradually improved after 61 cases
			Familiarization with port placements and robotic docking		Familiarization after 43 cases
rRS^a^ (rTARUP + eTEP)	Kudsi (16)	Retrospective CUSUM analysis	OT	C	OT Decrease after 29 cases
			Adverse outcome		Adverse outcomes decrease after 51 cases
rTARUP	Muysoms (17)	Prospective study	Study group of 41 cases	C	Decrease in overall OT related to improved efficiency in the dissection phase
eTEP with bilateral TAR	Douissard (18)	Abstract	Successful completion	C	Successful completion after 40 cases
		Retrospective study			
Bilateral TAR	Halpern (19)	Retrospective study	OT	C	Slow decline and less variation in OT with increased experience
	Kudsi (20)	Retrospective CUSUM analysis	Console time	C	Learning curve was overcome between 49 and 75 cases
			Postoperative complications		

OT, operative time; SSE, surgical site event.

^a^ Robotic-assisted retrorectus Rives-Stoppa repair.

### Ventral Hernias

Douissard et al. (6) highlight the importance of good patient selection for the initial cases when using the robotic platform for ventral hernias. They proposed that a good compromise between patient-centred approach in respect of the learning curve could be to select obese patients with symptomatic mid-line primary hernias over 2 cm. These patients, traditionally treated by an intraperitoneal mesh technique (IPOM), could indeed benefit from defect closure and extra-peritoneal mesh positioning offered by the robotic approach, while allowing skills acquisition in the robot assisted approach for the surgeon. Indeed, one of the drivers for surgeons to adopt RAWS is to perform extraperitoneal ventral hernia repair and avoid IPOM repair. Intra-peritoneal mesh placement is implicated in potential adverse events during subsequent abdominal procedures because of adhesions (13, 21). But extraperitoneal ventral hernia repair with conventional laparoscopic instruments is generally more challenging and difficult to perform (22). For this, using the technological advantages of the robotic platform seems ideal to overcome the challenges in performing extraperitoneal ventral hernia repair minimal invasively. Nevertheless, robot assisted laparoscopic IPOM repair has also been studied and has the advantage of a low complexity in anatomical dissection for the procedure, making it a good initial case choice for starting with RAWS in ventral hernias. Kudsi et al. used a CUSUM analysis and found a consistent and gradual decrease in OT after the completion of 36 cases and a risk-adjusted CUSUM revealed improving outcomes for complications after 55 cases (*trainee type C*) (23).

The next technique with low anatomical complexity is a preperitoneal repair for ventral hernias (rTAPP ventral) which also seems a good option for initial case selection for RAWS of ventral hernias. It avoids the intraperitoneal placement of mesh and allows for skills acquisition with the robotic platform in a delicate preperitoneal dissection around the hernia but avoiding the more complex anatomical challenges of retrorectus repair or robotic eTEP procedures. Kudsi et al. (14) investigated the learning curve of rTAPP ventral for primary ventral hernias in a CUSUM analysis. To achieve a steadily decreasing OT, 46 cases were needed whereas a good quality repair, defined through maintenance of the integrity of the peritoneal flap, gradually improved after 61 cases. Familiarization with the port placements and robotic docking was accomplished after 43 cases (*trainee type C*). After mastering robotic-inguinal and robotic small ventral hernia repairs, surgeons may take on larger defects with retromuscular approaches which may include component separations for tension free closures.

A robot assisted retrorectus Rives-Stoppa repair (rRS ventral) was studied again by Kudsi et al. with a CUSUM analysis and they found a consistently decreasing OT and adverse outcome rate after completion of 29 and 51 cases respectively. Both, a transabdominal approach (rTARUP) as a totally extraperitoneal access (eTEP) were used for the retrorectus repairs (*trainee type C*) (15). Muysoms et al. (16) analysed the OT for 41 cases of rTARUP and observed a decrease in overall OT that was mainly related to the improved efficiency in the dissection phase of the procedure. The technique is reproducible and safe, and the OT equalizes favourably with published OTs for laparoscopic and open retromuscular umbilical hernia repairs. Because the case series was performed after having reached proficiency with rTAPP for groin hernias no more time gain with the preparation or docking of the robotic platform was observed (*trainee type C*). An overview of the studies is listed in Table 2.

### Posterior Component Separation: Robotic TAR

Transversus abdominus release (TAR) in patients with ventral hernias is one technique to aid closure with acceptable tension of the posterior and anterior fascia. It also allows mesh positioning lateral to the confines of the rectus sheath i.e., retrorectus medially and retromuscular laterally. Performing a safe and effective TAR requires a good working knowledge of the abdominal wall anatomy with understanding of the different abdominal compartments. These operations were traditionally performed by open techniques, but a robotic TAR is perhaps one of the greatest benefits of RAWS, with significantly shortened hospital stay and return to activities (17, 24). These complex operations can be performed with conventional laparoscopic instruments but are surgically challenging (25). The technological advantages of the robotic platform facilitate these complex operations. The more complex the case, perhaps the more potential benefit of using the robotic platform in terms of reduced surgical stress. For many surgeons starting a RAWS program, the main driver is to handle more complex cases with significant patient benefit (6). But it is important to realize that two requirements are essential. Firstly, for *hernia specialists* the use of the robotic platform must have reached proficiency with less complex cases (*trainee type A or B*) and secondly, for robotic proficient *non-hernia specialists* (*trainee type F*) additional training on the anatomical specifics of the abdominal wall and TAR procedure is mandatory. Douissard et al in an abstract during the recent Annual European Hernia Society congress in Copenhagen described that successful completion of an eTEP robotic ventral hernia repair with bilateral TAR was achieved after 40 cases of ventral hernia repair (26). Halpern et al. described their learning curve for robotic abdominal wall reconstruction to achieve proficiency in performing a bilateral TAR (18). They saw a slow decline and less variation in OT with increased experience and highlighted the gradual progression of case complexity in their case selection from robotic transabdominal retrorectus umbilical prosthetic hernia repair (rTARUP) over unilateral robotic TAR to bilateral TAR. Kudsi et al. described their learning curve of robotic TAR in ventral hernia repair (19). This study revealed that the robot TAR learning curve was overcome between 49 and 75 cases, regardless of bilateral or complex status, after which, console time and postoperative complications decreased significantly. They also reported significantly less postoperative complications in the late phase as compared to the early phase, despite the increase in hernia complexity across the two study phases. The authors also commented that since this procedure can be considered an extension of robotic retrorectus repair, proficiency with robotic retrorectus repair must be reached prior to attempting rTAR (19). In our own experience a gradual decrease of OT is achieved, and OT reaches levels within the “comfort zone” of the surgeons, OR nurses, OR planners and anaesthetists after 20 to 25 cases (24). These cases can take many hours in the OR to begin with. It is important to educate the OR team and indeed hospital management that the benefits of the robotic approach benefits patients even in the early part of the learning curve. This was also stressed by (20). Indeed, for most surgeons reaching experience with 20 robotic TAR operations will take a period of 12 months or more considering the frequency of indications for TAR.

## The Technology Training Pathway for DaVinci Robot Assisted Surgery

**FIGURE 1:**
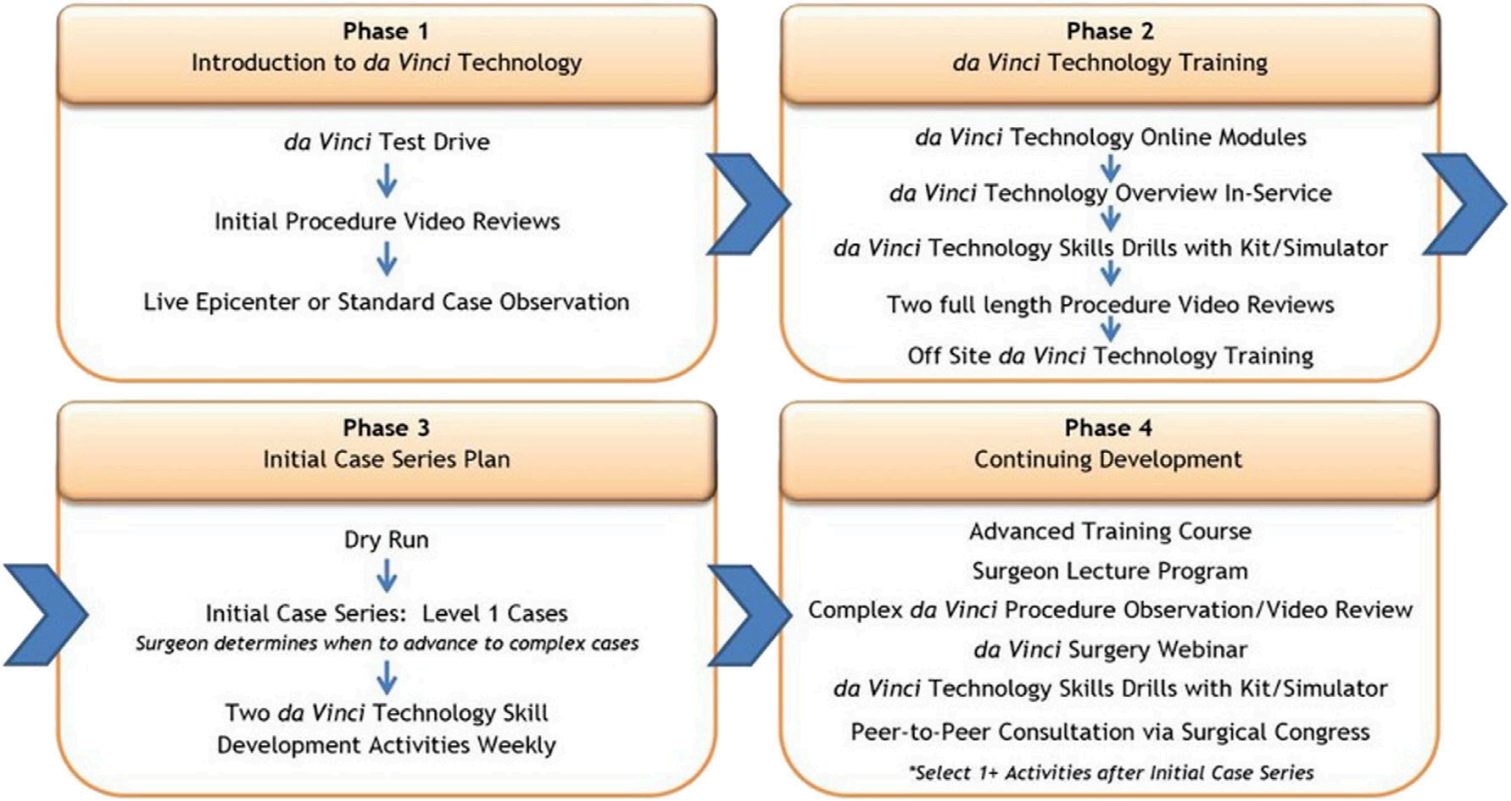
The 4 phases of the Intuitive training pathway: phases I and II are preclinical, while phases III and IV are the introduction of robot assisted surgery in clinical practice.

The Intuitive training pathway is constructed of 4 phases; phases I and II are preclinical, while phases III and IV are the introduction of robot assisted surgery in clinical practice (Figure 1). The specific training courses have been given names TR100, TR200, TR300 and TR 400, which the trainees must follow not only during the start of a robotic program, but also during the later progression towards more complex surgical procedures. These phases of the training program and the training activities are more generic and are not specific for RAWS. While they are used across several specialties, they can incorporate specific aspects of RAWS at each step.

### Phase I: Introduction to Robotic Technology and Hernia Surgery (Preclinical Phase)

In the first phase, surgeons aspiring to introduce a RAWS program will take part in a first test drive with the robotic platform. This often takes place during a visit at a medical conference booth or at one of the training centres. A concrete set-up plan should be established to ensure access to a robotic platform. This allows the surgeon to effectively build regular clinical experience in the first weeks after starting their clinical start-up program. By either attending a webinar by a robotic hernia proctor on how to set up a robotic hernia surgery program and/or by attending a clinical case observation or clinical insight, the trainee can see the value of RAWS for patient care while learning the steps needed to acquire skill proficiency. An “epicenter” refers to an internal Intuitive terminology and describes a more complex, more advanced case observation site, which has published own clinical data and has strong support and commitment form administration. Compared to a “standard” case observation site, an epicenter provides more details to the visiting surgeons from a holistic point of view—clinical and economical (Figure 1). It will provide the surgeon with more information and knowledge to gain the confidence of colleagues and the hospital management to advocate the value of developing a RAWS program in their hospital centre.

### Phase II: Training in Robotic Technology (Preclinical Phase)

With secured access to the robotic platform at their hospital centre, the trainee will start the second phase of the training program. In this phase the surgeon will gain knowledge of the robotic technology as preparation to become a console surgeon. At the same time, in-service technical training will be supported by the company’s local representative to train the nursing staff and bedside assistants to gain proficiency on how to handle the robotic platform. Online modules must be followed, and tests need to be completed successfully to receive certification as a console surgeon.

The trainee will perform technology skills acquisition and simulator training, including specific SimNow exercises for performing rTAPP groin hernia repair. It is recommended that the trainee completes about 20 h of simulator training or achieves minimal SimNow proficiency scoring before proceeding towards the next step of the training program, which is the TR100 and TR 200 training.

Phase II also includes reviewing surgical videos as well as analysing the specific procedure cards (rTAPP, rTARUP) with information on operating room set up, trocar placement and procedural steps. Confirmation of completion of all above Phase II prerequisites is required prior to course attendance.

In phase II, the trainee will visit a training centre for a 2-day basic training course. The first day (TR100) is hernia specific focused technology training by an Intuitive trainer. The second day (TR200) is surgeon led procedural training. The TR200 will be led by a surgeon who is an Intuitive proctor and will allow the trainee to learn the specifics of robotic TAPP for groin hernia, robotic TAPP for ventral hernias and robotic IPOM. The port placement, docking and procedural steps will be trained on either a cadaveric model, a porcine model, or a synthetic model. The timing of the TR200 course should be as close as possible to the start of the clinical phase of the trainee. Ideally the surgeon-proctor teaching at the TR200 course will also be the on-site proctor for the first clinical cases of the trainee in their hospital.

### Phase III: Initial Case Series Plan (Initial Clinical Phase)

Soon after the TR200 completion, the trainee should start the initial case series assisted by a proctor. The patients chosen for the initial case series must be uncomplicated hernia repairs with low anatomical complexity. Ideally, one gives preference to these operations where the trainee has already a good experience in performing the procedures with conventional laparoscopic approaches. This will allow the trainee to focus on performing the surgery with the novel robotic platform without the need to focus on anatomical or procedural challenges. By choosing low complex cases with a relatively short OT, the trainee can plan several cases during their first robotic surgical days and thus train consecutively on the set-up, the port placement, and the docking with their team.

Robotic groin hernia repair rTAPP is the ideal indication for this initial case series. Alternatively robotic TAPP ventral or robotic IPOM repair are good choices because they hold a low complexity in the anatomical and procedural steps. We recommend performing 30 cases with these indications before moving forward towards more complex cases after attending an additional TR300 course. It is recommended to secure access to the robotic platform for RAWS on a weekly basis to go through these 30 initial cases smoothly without a major interruption of clinical practice with the robotic platform. Recommended indications and patient characteristics for the initial case series are uncomplicated groin hernias, small to medium sized primary ventral hernias (umbilical and epigastric hernias, 2–4 cm wide) avoiding recurrences or hernias in patients with very high BMI.

### Phase IV: Continuing Development and Advanced Training (Advanced Clinical Phase)

During an advanced course TR300 in a training centre a surgeons-led training will focus on technical and anatomical aspects of more advanced RAWS procedures. A good technique to allow the use of the robotic platform for more difficult anatomic features includes robotic retrorectus ventral hernia repair, rTARUP. Trainees can attend a more advanced case observation in a centre for more complex robotic procedures prior to the TR300. Prior to the training, the trainee should review videos and specific procedure cards. The training will be performed in a cadaveric lab, a porcine model or synthetic model and includes specifics about set-up, trocar placements and anatomical dissection. The initial clinical cases after the TR300 should also be proctored either on-site or *via* tele-mentoring.

Additional more advanced procedures can follow the same training with a surgeon-led training for specific techniques like robotic eTEP or rTAR (TR400) on cadaveric lab or model. Major focus will be on anatomical challenges for these procedures. Also, the first rTAR should benefit from the attendance of a proctoring on-site or with remote tele-mentoring.

**FIGURE 2:**
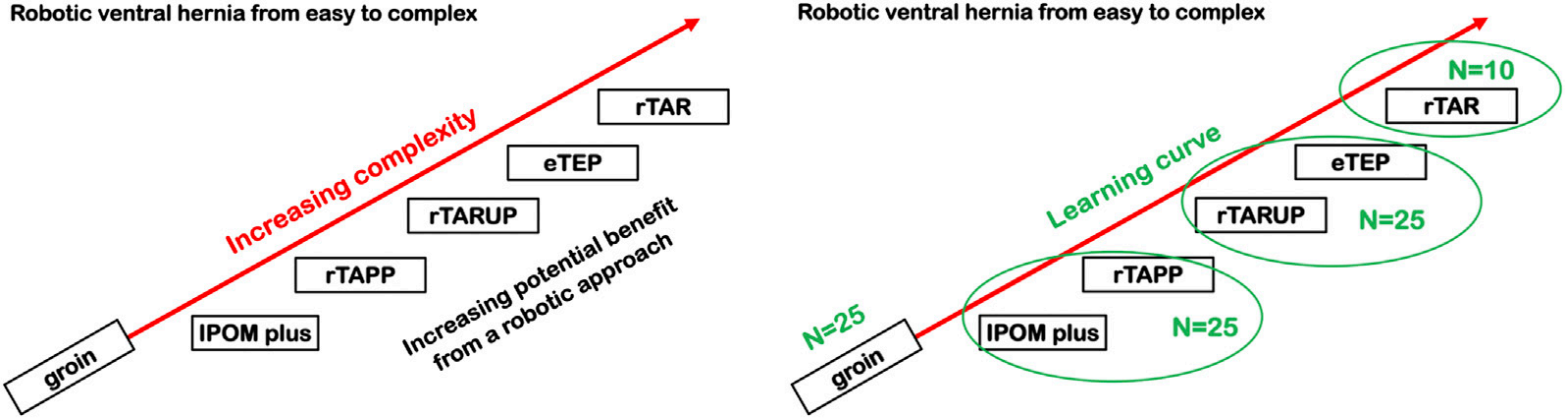
Proposal of a gradual increase in case complexity during the introduction of a RAWS training program. IPOM, Intraperitoneal Onlay Mesh; rTAPP, Robotic assisted Transabdominal preperitoneal prosthetic inguinal hernia repair; rTARUP, Robotic Transabdominal Retrorectus Umbilical Prosthetic hernia repair; eTEP, Enhanced Totally Extraperitoneal hernia repair; rTAR, Robotic assisted Transversus Abdominis Release.

In summary, it is recommended to follow a structured training approach with dedicated technology training followed by an initial case series that allows the trainee to develop surgical skills with the robotic platform. Gradual increase of case complexity will allow for increasing technical skill on the robotic platform whilst ensuring patient safety. The presence of a surgeon-proctor during the initial cases is a current standard of care (Figure 2).

The training pathway can be modified to the prior experience of the trainee surgeon. Surgeons with experience with robotic surgery for non-hernia indications (*trainee type C or F*), can start directly with phase II since technology training is no longer needed. Surgeons in training that are working in a department performing RAWS can be trained during their residency as part of their regular training program in the clinical phase. But the preclinical phase (phase I and II) should be undertaken by them prior to the start of clinical training.

**FIGURE 3:**
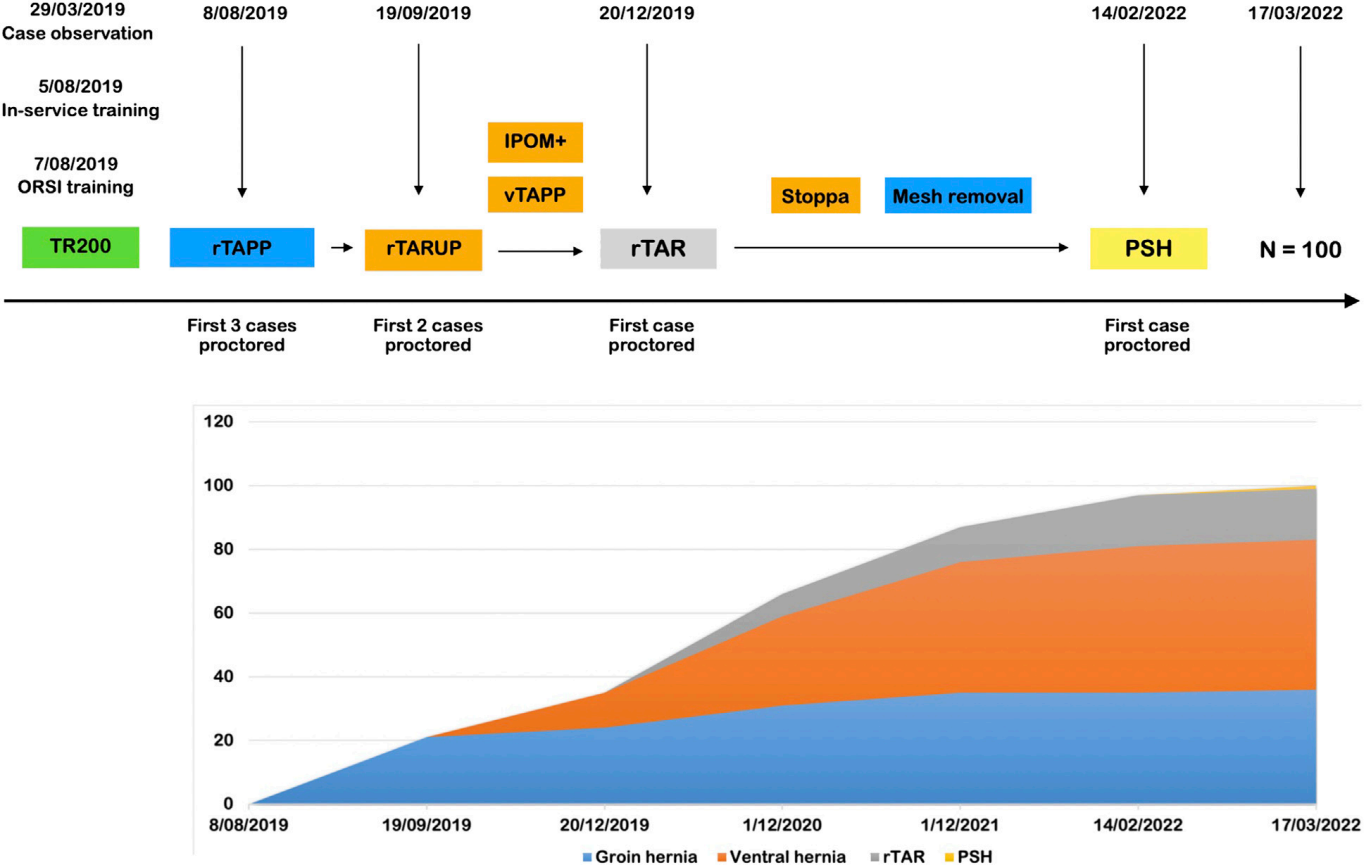
First 100 cases during the successful implementation of a RAWS program at the centre of one of the authors (MS). rTAPP, Robotic assisted Transabdominal preperitoneal prosthetic inguinal hernia repair; rTARUP, Robotic Transabdominal Retrorectus Umbilical Prosthetic hernia repair; IPOM, Intraperitoneal Onlay Mesh; vTAPP, Transabdominal preperitoneal prosthetic ventral hernia repair; rTAR, Robotic assisted Transversus Abdominis Release; PSH, Parastomal Hernia repair.

In Figure 3 we depict the implementation of a RAWS program at the centre of one of the authors (MS), a hernia specialist without prior experience in robotic surgery (trainee type B). In the preclinical phase (Phase I and II), the surgeon attended a clinical case observation which got the surgeon interested in building a RAWS program. After securing access to the robotic platform and planning the start-up, 5 months later, a technology training in the hospital for the whole OR team was followed, in addition to TR100 and TR200 training at the ORSI training centre in Belgium. This training was preceded by obtaining the online console certificate and intensive simulator training (25 h). The trainer at the TR200 was also the proctor for the first robotic groin hernia cases in the same week. After performing 20 cases of noncomplex groin hernias robotically, two cases of rTARUP were performed with a second proctor. As agreed with the hospital management after the initial learning curve with noncomplex groin hernia repairs, these operations were no longer performed using the robotic platform. Subsequently, ventral hernia repairs, mostly rTARUP, but also some IPOM cases and rTAPP ventral operations were performed. Also, some cases of complex groin hernias were still performed robotically for the added value of using the robotic technology. Later in the learning curve, which has unfortunately been hampered by the restrictions and limitations in surgical procedures during the COVID pandemic, the robotic platform was used for removal of preperitoneal mesh where the robotic technology seems to have good value. After having performed more than 40 ventral hernia cases robotically the first robotic TAR was planned in the presence of the first proctor. Subsequently, the first case of robotic parastomal hernia repair was performed with the second proctor concluding the apprenticeship. The surgeon has now become a proctor and trainer having performed more than 100 cases of RAWS.

## Discussion: Role of the European Hernia Society

It is evident that Intuitive takes the introduction of their robotic technology into a hospital seriously. And as far as they can, seek to ensure that the surgeon is appropriately trained and mentored in the learning curve. However, they are a medical robot company, and not a doctor certification authority. Hence, their partnership with renowned medical organizations is an important part of ensuring patient safety. A number of medical societies have taken up part of the training tasks through their robotic training curriculum. In the US, the American College of Surgeons (ACS) is providing a Fundamentals of Robotic Surgery (FRS) skills curriculum which has been shown to be effective in improving performance prior to the clinical adoption of robotic surgery (27). In Europe the European Association of Urology (EAU) has provided a robotic training curriculum that allows trainees a safe and effective adoption of robotic surgery and provides a certification on successful completion of the training program (28).

To date, the training of surgeons adopting RAWS utilizing the Intuitive robot platform, is managed by the Company. The EHS is involved in organizing courses outside of the official pathway for their members. These are discovery courses that fit into the preclinical phase of the training pathway for surgeons with no experience with robotic surgery but with aspiration of adopting RAWS into their practice. We do think the preclinical phase of technology training is mainly a responsibility of the company providing the robot platform. But for the more clinical training, the EHS should play an important central role. It should provide a training curriculum similar to what the EAU has provided and a FRS course similar to what is offered by the ACS. This could lead to a certification of surgeons of having followed the training pathway for RAWS. However, who funds such courses, the EHS, the trainee, the hospitals or the robot company is unclear.

A dedicated EHS course on the specific anatomy of the abdominal wall and procedural steps of performing complex abdominal wall reconstruction like TAR, would certainly fall within the expertise and mission of the EHS. These courses should not be specific to robotic surgery but would be very helpful in ascending the learning curve of RAWS towards the more complex cases.

Quality control is certainly another mission of the EHS, and we provide an online registry that can capture data on all hernia operations which could be a valuable tool for collection of data and follow up. Indeed, such data collection should be mandated to allow good assessment of the benefits and risks of RAWS, as it moves from a rare surgical technique in many countries into mainstream practice. Outcomes can be quantified and if enough surgeons contribute, one might bench mark its clinical practice against others as well as compare different operative approaches.

## Robotic Surgery Task Force of the European Hernia Society

Andrew de Beaux (General secretary of the EHS); Maarten Simons (President elect of the EHS); Barbora East (Secretary of Quality of the EHS); Cesare Stabilini (Secretary of Science of the EHS); Wolfgang Reinpold (Past Secretary of Education of the EHS); Knut Borch (Secretary of Education of the EHS); Filip Muysoms (President of the EHS).

## Footnotes

1
https://uemssurg.org/wp-content/uploads/2021/07/AW-Eligibility-requirements.pdf

## Abbreviations

ACS, American College of Surgeons; (R)AWS, (Robotic) Abdominal Wall Surgery; CUSUM, Cumulative Sum Analysis; EAU, European Association of Urology; EBS, European Board of Specialists; EHS, European Hernia Society; eTEP, Enhanced Totally Extraperitoneal hernia repair; FRS, Fundamentals of Robotic Surgery; GEARS, Global Evaluative Assessment of Robotic Skills; IPOM, Intraperitoneal Onlay Mesh; OT, Operative Time; SSE, Surgical Site Event; PSH, Parastomal Hernia; rRS, Robotic assisted retrorectus Rives-Stoppa repair; rTARUP, Robotic Transabdominal Retrorectus Umbilical Prosthetic hernia repair; (r)TAPP, (Robotic assisted) Transabdominal preperitoneal prosthetic inguinal hernia repair; (r)TAR (Robotic assisted) Transversus Abdominis Release; TEP, Totally extraperitoneal prosthetic inguinal hernia repair; UEMS, Union of European Medical Specialists; vTAPP, Transabdominal preperitoneal prosthetic ventral hernia repair.
